# Ocular surface disorders affect quality of life in patients with autoimmune blistering skin diseases: a cross-sectional study

**DOI:** 10.1186/s12886-022-02663-w

**Published:** 2022-11-15

**Authors:** Huanmin Kang, Mengbo Wu, Jianing Feng, Yuerong Ren, Yingyi Liu, Wen Shi, Yingqian Peng, Yixin Tan, Ruifang Wu, Guiying Zhang, Yan He

**Affiliations:** 1 Department of Ophthalmology, The Second Xiangya Hospital, Central South University, Changsha, 410011 Hunan China; 2 Hunan Clinical Research Center of Ophthalmic Disease, Changsha, Hunan China; 3 Department of Dermatology, The Second Xiangya Hospital, Central South University, Changsha, Hunan China

**Keywords:** Ocular surface, Autoimmune skin diseases, Dry eye, Quality of life, Questionnaire

## Abstract

**Background:**

Autoimmune blistering skin diseases (AIBD) are a group of rare chronic autoimmune diseases which are associated with ocular surface diseases especially dry eye disease. This study is designed to investigate the relationship between ocular surface disorders and quality of life among patients with autoimmune blistering skin diseases.

**Methods:**

Twenty-four AIBD patients (18 pemphigus and 7 pemphigoid) and twenty-five non-AIBD controls were included. Ocular surface disease index (OSDI), ocular surface evaluation, including slit-lamp examination, Schirmer I test, tear break-up time, corneal fluorescein staining, lid-parallel conjunctival folds, meibomian gland evaluation, presence of symblepharon and corneal opacity were assessed. Life quality was evaluated by multiple questionnaires, including Medical Outcomes Study 36-Item Short Form Questionnaire (SF-36), Hospital Anxiety and Depression Scale (HADS), Pittsburgh Sleep Quality Index (PSQI) and Health Assessment Questionnaire-Disability Index (HAQ-DI). Ocular surface tests and quality of life were compared between AIBD patients and non-AIBD controls. In the AIBD patients, the associations between ocular surface parameters and quality of life were also evaluated.

**Results:**

92% of AIBD patients and 87.5% of age- and sex-matched non-AIBD controls were diagnosed with dry eye in this study. Compared with non-AIBD controls, AIBD patients reported lower SF-36 scores (*P* < 0.05) and severer OSDI, Schirmer I test, tear break-up time, corneal fluorescein staining, presence of symblepharon and corneal opacity measures (*P* < 0.05). OSDI, Schirmer I test were correlated with SF-36 composite scores or scores on the SF-36 subscales.

**Conclusions:**

AIBD patients experience reduced quality of life and more severe ocular surface disorders including dry eye, symblepharon and corneal opacity. Early treatments of dry eye and collaborations among multidisciplinary physicians are necessary in patients with AIBD.

## Background

Autoimmune blistering skin diseases (AIBD) are a group of rare chronic autoimmune disorders, mainly including pemphigus and pemphigoid. They are characterized by the cohesion disruption of intraepidermal (pemphigus) or subepidermal (mainly pemphigoid), which could lead to erosions on the skin and/or the mucous membranes [1]. The common subtypes of pemphigus are pemphigus vulgaris (PV), pemphigus foliaceus (PF), and paraneoplastic pemphigus (PNP). Pemphigoid mainly has 3 forms: bullous pemphigoid (BP), mucous membrane pemphigoid (MMP), and epidermolysis bullosa acquisita (EBA) [2].

AIBD involve a broad range of clinical presentations, including ocular disorders, neurological diseases, gastrointestinal inflammation, rheumatoid arthritis, autoimmune thyroid disease and type I diabetes [3, 4]. When the ocular complications in patients with AIBD are mentioned, most ophthalmologists think of ocular cicatricial pemphigoid (OCP), a subtype of MMP [5]. Previous clinical observations have reported that many patients with other AIBD subtypes also present various ocular disorders that affect quality of life but often go underrecognized and undertreated [6]. Jeremy C.K. Tan et al. found that 77.3% of patients with PV or BP complained of at least one ocular symptom related to dry eye [7], which might have relevance to the progression and medications of disease.

Dry eye is a common multifactorial ocular surface disease characterized by a loss of homeostasis of the tear film combined with various ocular symptoms, including pain, irritation, light sensitivity, foreign-body sensation, dryness, and fluctuating vision [6]. The prevalence of dry eye varies in different areas, but the number of affected individuals is increasing [6]. Its primary mechanism is evaporative water loss leading to tissue damage, which could further lead to the loss of epithelial cells and goblet cells directly or by inducing inflammation [8]. Psychiatric disorders, anxiety, pressure and reduced sleeping quality have been reported to be risk factors for dry eye [9, 10]. However, only a few studies have focused on their relationship in some rare immunological diseases, including AIBD.

Obstruction in the lacrimal ducts, meibomian gland dysfunction (MGD), increased tear osmolality (TO), symblepharon, conjunctival erosion, corneal opacity and other pathological changes in patients with AIBD may cause dry eye [8]. Additionally, the symptoms that cause a decrease in quality of life, including psychiatric disorders, anxiety, pressure and low sleep quality, may also trigger and worsen dry eye in patients with AIBD, which are underestimated with limited reports. We therefore carried out a clinical study evaluating ocular manifestations, especially dry eye, in patients with AIBD and their quality of life by using reliable and valid self-report questionnaires. These questionnaires included the Ocular Surface Disease Index (OSDI) [11], Medical Outcomes Study 36-Item Short Form (SF-36) [12], Hospital Anxiety and Depression Scale (HADS) [13], Pittsburgh Sleep Quality Index (PSQI) [14], and Health Assessment Questionnaire-Disability Index (HAQ-DI) [15]. The OSDI is an efficient instrument for measuring the effect on vision-related function and dry eye disease. As an indicator of overall health status, the SF-36 is designed to obtain patients’ perception of their well-being and health. The HADS aims to detect states of depression and anxiety. The PSQI is an effective tool for assessing the quality of sleep in patients. Considering the increasing prevalence of RA in patients with AIBD [4], the HAQ-DI was also implemented in this study, which is a comprehensive measure of outcome in patients with rheumatic diseases.

This investigation aims to detect the quality of life, states of depression and anxiety, sleep quality and severity of ocular surface disorders in patients with AIBD, and to explore the relationship between dry eye and quality of life in patients with AIBD.

## Methods

### Subjects

In this study, 25 AIBD patients and 24 non-AIBD volunteers were recruited from March to July 2021 with informed consent. The research was approved by the institutional human experimentation ethics committee of the Second Xiangya Hospital (code of ethics: LYF2021028) and adhered to the Tenets of the Declaration of Helsinki. All patients were diagnosed with AIBD by skilled dermatologists and voluntarily participated in this study after signing the informed consents, but they were not restricted to referrals by dermatologists. Those non-AIBD subjects without already known dry eye disease served as non-AIBD controls. All participants are required to have no difficulty in communication and be older than 18 years according to the inclusion criteria.

### Ocular surface evaluation

Available diagnostic tests on eyes, including the Schirmer I test without anesthesia, tear break-up time (TBUT), cornea fluorescein staining (Oxford grading scheme [16]), slit lamp assessment of the lid margin (LIPCOF grading) and meibomian gland secretion grading [17], were implemented by adequately trained ophthalmologists.

Schirmer I test ≤ 5 mm/ 5 min, TBUT ≤ 10 s, cornea fluorescein staining Oxford grade > 0, or LIPCOF (lid-parallel conjunctival folds) grade ≥ 2 were considered abnormal. Dry eye is a multifactorial disease and there is no single gold standard sign or symptom that match the state of dry eye. In our study, when patients have dry eye symptoms (OSDI ≥ 13) and meet one of the above abnormal signs can be diagnosed by dry eye disease. Considering the non-proportionality of symptoms and signs, when patients were asymptomatic or minimally symptomatic, while there was severe damage on the tear function or ocular surface which are unattributable to other specific conditions, can be also diagnosed with dry eye disease [17]. The severity of dry eye was assessed according to the dry eye severity grading scheme as reported [18]. The level is evaluated by the severity of signs and symptoms used to guide the therapy.

### Questionnaires

When these ocular tests were administered, questionnaires such as the OSDI, SF-36, HADS, PSQI, and HAQ-DI were completed by patients after basic instructions were provided.

The OSDI questionnaire is composed of 12 vision-targeted questions sorted by 3 parts: ocular symptoms, environmental triggers and vision-related function. The scores are calculated using the following formula: OSDI = (sum of scores for all questions answered × 100)/ (total number of questions answered × 4). Higher scores indicate more severe conditions, and the overall OSDI scores define the ocular surface as normal (0–12), mild (13–22), moderate (23–32) or severe (above 33) [17].

The SF-36 is a 36-item questionnaire that consists of eight subscales: physical functioning, physical role functioning, bodily pain, general health perceptions, vitality, social role functioning, emotional role functioning and mental health. The total score is calculated using the weighted sums of the questions in each section and ranges from 0–100. Moreover, the physical component summary (PCS) and mental component summary (MCS) are aggregated from the 8 scaled scores and calculated as previously reported [19]. Higher scores on the SF-36 indicate less disability.

The HADS contains 14 items (7 anxiety and 7 depression), and each item was answered by the objects on a 0-3 severity scale, so the possible scores range from 0-21 for anxiety or depression. The analysis of scores is also based on two subscales. A score of 0 to 7 is regarded as normal, a score of 8 to 10 is considered to indicate the probable presence of the respective state, and a score of 11 or higher is indicative of a mood disorder [20].

The PSQI has 7 subscales: subjective sleep quality, sleep latency, sleep duration, habitual sleep efficiency, sleep disturbances, use of sleeping medication, and daytime dysfunction over the last month. Scores on each subscale range from 0 to 3, and thus, the total score of the index ranges from 0 to 21, with higher scores indicating worse sleep quality. A total score of 5 or greater indicates poor sleeping quality [21].

The HAQ is an effective tool for assessing disability across seven components: dressing, arising, eating, walking, hygiene, reach, grip, and activities. There are 2 or 3 questions for each component, and the response options for each item range from 0 (without any difficulty) to 3 (unable to do). For each component, the score is determined by selecting the worst score on any item. The sum of each component is then divided by the number of components answered, and the total score ranges from 0-3 [22].

### Statistical analysis

The statistics are presented as the mean and standard deviation (SD) for continuous measurements and as the number and percentage of total for categorical measurements. Comparisons of parametric data were performed with independent samples t-tests. The rank sum test was used to assess the rank data and nonparametric data differences between AIBD and the non-AIBD controls. The counting data were analyzed by the chi-square test or Fisher’s exact test to detect the significant differences between the two groups. The correlation between the questionnaire results and patients’ dry eye parameters was evaluated using the Pearson correlation coefficient or Spearman rank correlation for nonnormally distributed data and rank data. Statistical analysis was performed with SPSS 20 (SPSS Inc., Chicago, IL, USA), and a two-sided *P* value of < 0.05 was considered statistically significant for all reported tests.

## Results

### Characteristics of participants

Fifty eyes of 25 AIBD patients (11 female and 14 male) were included, with an average age of 55.16 ± 12.69 years (range from 32 to 79 years old). Among them, 18 patients (72.0%) were diagnosed with pemphigus (16 PV, 2 PF), and the remaining 7 patients (28.0%) were diagnosed with pemphigoid (4 MMP, 3 BP). There was 1 female diagnosed with OCP. Forty-eight eyes of 24 non-AIBD volunteers (14 female and 10 male) ranging from 23 to 77 years old were also enrolled in this study. No statistically significant differences in age or sex were observed between the AIBD group and the non-AIBD control group. The demographic data and other characteristics of AIBD patients and non-AIBD volunteers are shown in Table 1.

**Table 1:** The basic clinical information and ocular surface parameters of AIBD patients and non-AIBD controls

Characteristics	AIBD patients	Non-AIBD controls	*P* value
**Subjects (n)**	25	24	
**Age (years)**	55.16 ± 12.69	53.25 ± 16.31	0.649
**Gender, n (%)**			0.316
Male	14 (56.0%)	10 (36.0%)	
Female	11 (44.0%)	14 (64.0%)	
**OSDI score**	36.25 ± 25.33	21.29 ± 18.63	**0.023**
**OSDI level, n (%)**			**0.029**
0 (0–12)	6 (24.0%)	11 (45.8%)	
1 (13–22)	4 (16.0%)	5 (20.8%)	
2 (23–32)	0 (0.0%)	2 (8.3%)	
3 (≥ 33)	15 (60.0%)	6 (25.0%)	
**Eyes (n)**	50	48	
**Schirmer I test (mm/5 min)**	8.42 ± 4.86	12.50 ± 9.00	**0.006**
**Schirmer I test level, n (%)**			0.594
≤ 5 mm/5 min	16 (32.0%)	13 (27.1%)	
> 5 mm/5 min	34 (68.0%)	35 (72.9%)	
**TBUT (s)**	5.68 ± 3.37	7.42 ± 4.52	**0.033**
**TBUT level, n (%)**			< **0.001**
≤ 10 s	47 (94.0%)	31 (64.6%)	
> 10 s	3 (6.0%)	17 (35.4%)	
**Corneal fluorescein staining, n (%)**			< **0.001**
Normal (grade 0)	34 (68.0%)	46 (95.8%)	
Abnormal (≥ grade 1)	16 (32.0%)	2 (4.2%)	
**LIPCOF grade, n (%)**			0.147
0- No conjunctival fold	20 (40.0%)	29 (60.4%)	
1- One permanent and clear fold	8 (16.0%)	6 (12.5%)	
2- Two permanent and clear fold	11 (22.0%)	4 (8.3%)	
3- More than 2 permanent and clear parallel fold	11 (22.0%)	9 (18.8%)	
**Meibomian gland secretion, n (%)**			0.420
0- Clear fluid	20 (40.0%)	23 (48.9%)	
1- Cloudy fluid	23 (46.0%)	18 (38.3%)	
2- Granular and cloudy fluid	5 (10.0%)	6 (12.8%)	
3- Toothpaste like opaque or harder	2 (4.0%)	0 (0.0%)	
**Symblepharon presence, n (%)**			**0.004**
Normal	42 (84.0%)	48 (100.0%)	
Abnormal	8 (16.0%)	0 (0.0%)	
**Cornea clarity, n (%)**			**0.013**
Normal	44 (88.0%)	48 (100.0%)	
Abnormal	6 (12.0%)	0 (0.0%)	
**Dry eye level, n (%)**			**0.001**
No dry eye	2 (8.0%)	3 (12.5%)	
Grade 1	2 (8.0%)	11 (45.8%)	
Grade 2	3 (12.0%)	5 (20.8%)	
Grade 3	14 (56.0%)	5 (20.8%)	
Grade 4	4 (16.0%)	0 (0.0%)	

Continuous variables were compared using the independent t tests and shown as mean ± SD. Chi-square test was used for the comparison of categorical data. Mann–Whitney U test was used to compare ranks

*AIBD* Autoimmune blistering skin diseases, *TBUT* Tear break-up time, *OSDI* Ocular Surface Disease Index, *LIPCOF* Lid-parallel conjunctival folds

Significant differences (*P* value < 0.05) are bolded

### Ocular surface parameters

As a symptom severity assessment of dry eye, the OSDI questionnaire was completed by all participants, with scores ranging from 0 to 98. OSDI scores were > 22 in 15 (60.0%) AIBD patients, and the average OSDI score (36.25 ± 25.33) in patients was significantly higher than that in age- and sex-matched non-AIBD volunteers (21.29 ± 18.63, *P* = 0.023). A significant difference was also found in the OSDI level (0-3) between the two groups (Table 1; *P* = 0.029).

For the assessment of tear film, the Schirmer I test, TBUT test and corneal fluorescein staining were performed in all AIBD patients and non-AIBD volunteers. The mean Schirmer I test of the control group (12.50 ± 9.00 mm/5 min) was significantly higher than that of the AIBD patients (8.42 ± 4.86 mm/5 min; Table 1; *P* = 0.006). TBUT was abnormal in 94.0% of eyes of AIBD patients (range from 1 to 15 s) and 64.6% of non-AIBD volunteers (range from 2 to 20 s), with a significant difference (Table 1; *P* < 0.001). The corneal fluorescein staining Oxford score was ≥ grade 1 in 32.0% of AIBD eyes and higher than 4.2% in the non-AIBD controls (Table 1; *P* < 0.001).

In terms of ocular surface findings, statistically significant differences in symblepharon presence (*P* = 0.004) and cornea clarity (*P* = 0.013) were observed when comparing the AIBD group with the non-AIBD group (Table 1). Among 50 eyes of AIBD patients, symblepharon was present in 8 eyes (16.0%, from 2 females and 2 males), and corneal opacity was present in 6 eyes (12.0%, from 2 females and 1 male). Conversely, neither symblepharon presence nor corneal opacity was found in all non-AIBD volunteers (Table 1). The LIPCOF grade was abnormal in 44.0% of AIBD patients and 27.1% of control volunteers. There was no significant difference between the two groups (Table 1; *P* = 0.051). Additionally, 60.00% of eyes in patients with AIBD patients and 51.1% of eyes in the non-AIBD controls were found to have abnormal meibomian gland secretion; this difference was not statistically significant (Table 1; *P* = 0.376).

In this study, 92.0% of AIBD patients and 87.5% of non-AIBD controls were diagnosed with dry eye. Most of the AIBD patients (56.0%) had grade 3 dry eye, while a majority of age- and sex-matched non-AIBD volunteers (45.8%) had grade 1 dry eye. This difference was significant (Table 1; *P* = 0.001).

No statistical significance was detected when comparing the ocular surface parameters of pemphigus patients with those of pemphigoid patients.

### Questionnaire results

**Fig. 1:**
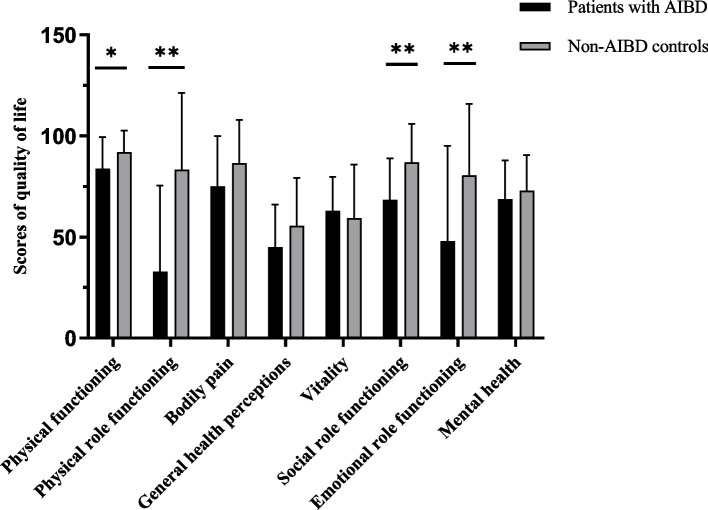
Comparison of SF-36 subitems results between patients with AIBD and non-AIBD controls. SF-36: Medical Outcomes Study 36-Item Short Form questionnaire; AIBD: autoimmune blistering skin diseases. * presents 0.01 < *P* < 0.05, ** presents *P* < 0.01. Bars represent the means; error bars represent the standard error of the mean

Four questionnaires (SF-36, HAQ-DI, PSQI and HADS) were administered. As shown in Fig. 1, among SF-36 subscales, AIBD patients had lower scores on all subscales except vitality (63.00 in patients with AIBD and 59.38 in the control group, *P* = 0.568); there were significant difference between groups in the scores on the physical functioning, physical role functioning, social role functioning and emotional role functioning subscales (each *P* < 0.05). Moreover, the MCS and PCS scores were significantly lower among AIBD patients than among the control patients (Table 2; *P* = 0.007, *P* < 0.001).

**Table 2:** Comparison of the questionnaires between AIBD patients and non-AIBD controls

	**AIBD patients**	**Non-AIBD controls**	***P* value**
	**(*n* = 25)**	**(*n* = 24)**	
**SF-36**	59.64 ± 17.32	79.04 ± 17.97	** < 0.001**
MSC	61.56 ± 19.78	77.54 ± 19.90	**0.007**
PCS	57.96 ± 20.56	80.88 ± 19.11	** < 0.001**
Physical functioning	83.80 ± 15.63	92.08 ± 10.62	**0.036**
Physical role functioning	33.00 ± 42.52	83.33 ± 38.07	** < 0.001**
Bodily pain	74.96 ± 24.98	86.58 ± 21.43	0.088
General health perceptions	45.00 ± 21.11	55.42 ± 23.82	0.112
Vitality	63.00 ± 16.77	59.38 ± 26.43	0.568
Social role functioning	68.28 ± 21.61	86.96 ± 19.03	**0.002**
Emotional role functioning	47.92 ± 47.21	80.50 ± 35.36	**0.009**
Mental health	65.20 ± 21.43	73.00 ± 17.47	0.170
**HADS**			
Anxiety	5.96 ± 3.40	3.75 ± 3.23	**0.024**
Depression	7.44 ± 4.00	6.13 ± 4.49	0.284
**PSQI**	6.68 ± 3.96	7.54 ± 4.45	0.478
**PSQI level, n (%)**			0.355
Normal (scores < 5)	9 (36.0%)	5 (20.8%)	
Abnormal (scores ≥ 5)	16 (64.0%)	19 (79.2%)	
**HAQ-DI**	0.16 ± 0.47	0.04 ± 0.20	0.264

*SF-36* Medical Outcomes Study 36-Item Short Form questionnaire, *PCS* Physical component summary, *MCS* Mental component summary, *HADS* Hospital Anxiety and Depression Scale, *PSQI* Pittsburgh Sleep Quality Index, *HAQ-DI* Health Assessment Questionnaire-Disability Index, *AIBD* Autoimmune blistering skin diseases

Significant differences (*P* value < 0.05) are bolded

As to HADS questionnaire, the average scores of anxiety and depression in the AIBD group were 5.96 and 7.44, respectively, and both scores were higher than those in the control group (3.75 and 6.13). There was a statistically significant difference in the anxiety score between the two groups (Table 2; *P* = 0.024).

In PSQI questionnaire, no significant difference in sleep quality was observed between the AIBD group and non-AIBD volunteers (Table 2; *P* = 0.478).

There was no significant difference in HAQ-DI scores between AIBD patients and non-AIBD controls (Table 2; *P* = 0.264).

Moreover, the analysis of completed questionnaires (SF-36, HAQ-DI, PSQI and HADS) showed that there was no significant difference between pemphigus and pemphigoid patients.

### The correlation between questionnaire results and ocular surface parameters in patients with AIBD

Pearson correlation analysis or Spearman rank correlation analysis was used to evaluate the association of the questionnaire results with dry eye parameters in all AIBD patients (*n* = 25).

**Fig. 2:**
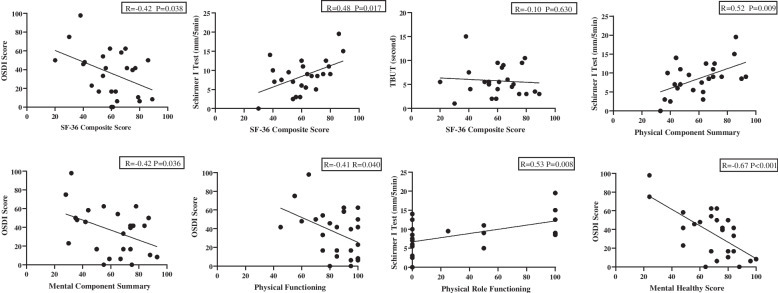
Correlations between SF-36 or subitems with dry eye symptoms and signs. OSDI: Ocular Surface Disease Index; TBUT: tear break up time; R: Pearson correlation coefficient. Higher scores in the SF-36 indicate better quality of life (note the negative correlation with OSDI, positive correlation with Schirmer I test)

There was a statistically significant correlation between the OSDI score and SF-36 composite score (Fig. 2; *R* = -0.42, *P* = 0.038). The OSDI score was also significantly correlated with MCS (Fig. 2; *R* = -0.42, *P* = 0.036), physical functioning (Fig. 2; *R* = -0.41, *P* = 0.040), metal healthy score (Fig. 2; *R* = -0.67, *P* < 0.001). However, there was no statistically significant correlation between OSDI scores and other questionnaire scores.

Among the ocular surface parameters, the Schirmer I test also showed a statistically significant correlation with the SF-36 composite score (Fig. 2; *R* = 0.48, *P* = 0.017) and some of the SF-36 subscale scores (Table 3 and Fig. 2). Moreover, other ocular surface parameters, including tear break-up time, cornea fluorescein staining and lid-parallel conjunctival folds, were not correlated with the questionnaire results, except meibomian gland dysfunction, which showed a mild but statistically significant correlation with the general health score (Table 3; *R* = 0.40, *P* = 0.049). There was no correlation between the questionnaire results and dry eye level.

**Table 3:** Correlations between questionnaires and ocular surface parameters in AIBD patients (*n* = 25)

	**OSDI**	**Schirmer I**	**TBUT**	**CFS**	**LIPCOF**	**MGS**	**DEL**
**SF-36 scales**							
Composite score	-0.42*	0.48*	-0.10	-0.08	0.04	0.07	0.06
PCS	-0.30	0.52**	-0.04	-0.26	-0.10	0.25	-0.04
MCS	-0.42*	0.30	-0.15	0.09	0.20	-0.17	0.09
PF	-0.41*	0.20	0.09	0.10	0.11	0.05	-0.13
RP	-0.20	0.53**	-0.10	-0.08	-0.20	0.09	0.04
BP	-0.17	0.21	0.07	-0.41	0.05	0.14	-0.12
GH	-0.14	0.35	-0.12	-0.08	0.00	0.40*	0.05
VT	-0.11	0.30	0.11	-0.19	0.04	0.08	-0.02
SF	-0.32	0.24	-0.23	-0.13	-0.01	-0.04	0.16
RE	-0.25	0.19	-0.13	0.25	0.28	-0.18	0.16
MH	-0.67**	0.23	-0.12	-0.03	0.19	-0.11	-0.16
**HADS**							
Anxiety	0.33	-0.25	0.21	-0.05	-0.15	-0.04	0.16
Depression	0.35	-0.01	0.37	-0.31	-0.29	-0.06	-0.07
**PSQI**	0.35	-0.32	0.25	0.04	0.12	-0.04	-0.08
**HAQ-DI**	0.27	-0.35	-0.17	0.39	0.04	-0.02	0.08

Correlations between two continuous variables were computed using the Pearson correlation coefficient, or Spearman rank correlation for non-normally distributed data and rank data

Higher scores in the SF-36 indicate better quality of life (note the negative correlation with OSDI, the positive correlation with Schirmer I test)

*AIBD* Autoimmune blistering skin diseases, *SF-36* Medical Outcomes Study 36-Item Short Form questionnaire, *PCS* Physical component summary, *MCS* Mental component summary, *PF* Physical functioning, *RP* Physical role functioning, *BP* Bodily pain, *GH* General health perceptions, *VT* Vitality, *SF* Social role functioning, *RE* Emotional role functioning, *MH* Mental health, *HADS* Hospital Anxiety and Depression Scale, *PSQI* Pittsburgh Sleep Quality Index, *HAQ-DI* Health Assessment Questionnaire-Disability Index, *OSDI* Ocular Surface Disease Index, *TBUT* Tear break-up time, *CFS* Corneal fluorescein staining, *LIPCOF* Lid-parallel conjunctival folds, *MGS* Meibomian gland secretion, *DEL* Dry eye level

^*^ Presents 0.01 < *P* < 0.05, ** presents 0.001 < *P* < 0.01, and the significant differences are bolded

## Discussion

AIBD are a group of chronic autoimmune disorders that are predominantly dermatologically present. In known clinical cases, a substantial proportion of AIBD patients complained of ocular involvements, such as irritation, pain, burning, decreased vision, discharge, corneal abrasion, conjunctival scarring and symblepharon [23]. Studies have also revealed that patients with AIBD can develop ocular complications, from mild dry eye disease, conjunctival inflammation, trichiasis and ocular surface scarring to severe corneal opacification and corneal perforation, which compromise the quality of life or even threaten vision [24–26].

Among these ocular involvements, dry eye presents as a typical complication and the early stage of ocular involvement [7]. It is a multifactorial ocular surface disease characterized by tear film instability [6]. The tear film consists of three layers, oil, aqueous and mucin, produced by meibomian glands, lacrimal glands or accessory lacrimal glands and conjunctival goblet cells, respectively. In present studies, dry eye in patients with AIBD may reflect a reduction in conjunctival goblet cells, fibrotic occlusion of the ducts of lacrimal glands and meibomian gland dysfunction, which result from progressive fibrosis, chronic inflammation, autoimmunity or drug use [27, 28]. A study involving 22 AIBD patients conducted by Jeremy C.K. Tan et al. revealed that a reduced Schirmer test was found in 92.9% of patients, and 100% of them had an abnormal TBUT with a median OSDI score of 10 [7]. Discordance between reported symptoms and observed signs in dry eye is commonly noted [6]. In our study, comprehensive dry eye assessments were implemented for all participants. We administered an OSDI questionnaire as well as TBUT and Schirmer I tests, and we performed corneal fluorescein staining, LIPCOF, meibomian gland secretion, presence of symblepharon and corneal opacity.

However, continuing evidence has indicated that dry eye is significantly associated with poor quality of life, anxiety, depression, psychiatric symptoms and impaired sleep quality, which also shows a high prevalence in patients with AIBD and leads to serious deleterious effects on quality of life [29, 30]. To date, only a few studies have measured subjective well-being in patients with AIBD, and no study has focused on poorer quality of life related to dry eye in patients with AIBD. The impact of dry eye and its role in patients with AIBD have not been clarified, while current studies suggest the possible relationship of dry eye with the quality of life in patients with AIBD. Therefore, we comprehensively assessed the quality of life in patients with AIBD by administering questionnaires such as the SF-36, HADS, PSQI and HAQ-DI to achieve a more holistic assessment of quality of life for patients with AIBD and to analyze its relationship between quality of life and dry eye in these patients.

In this study, compared with non-AIBD controls, AIBD patients were found to have lower quality of life as measured by the SF-36 questionnaire, and poor quality of life was associated with severer symptoms and signs (such as the Schirmer I test) in dry eye. The SF-36 is widely used to evaluate health-related quality of life, especially in cancer [31], chronic diseases [32, 33] and postoperative evaluation [34]. As a chronic disease, the SF-36 was also administered to patients with AIBD to evaluate their quality of life, particularly their psychological profile and fatigue [35, 36]. There were also statistically significant differences in HADS scores between AIBD and non-AIBD volunteers. Our study implies that early diagnosis, referral, and treatment of AIBD patients with dry eye is essential to improve their quality of life. Poor mental status and dry eye syndrome can both significantly decrease quality of life in patients with AIBD patients; in turn, poor quality of life can also aggravate AIBD symptoms, including dry eye. Therefore, it is important to pay attention to the quality of life and presentations of dry eye in the management of patients. Further studies are needed to clarify the directionality of this association and to determine how to alleviate dry eye symptomatology and improve quality of life in patients with AIBD patients.

There are some limitations in this study. First, due to low incidence of AIBD, our study is limited by the small sample size. Second is the single-center design, so the single-center effects can’t be excluded. Third, with the limit of our study, we did not comprehensively assess the impact of AIBD severity to dry eye. In clinical setting, it is possible that severity of AIBD could influence the manifestations of dry eye [37]. Finally, due to the non-representative nature of participants, the volunteer bias can’t be ignored.

## Conclusions

In conclusion, this study may show that a multidisciplinary collaboration between specialists in ophthalmology, dermatology, internal medicine and psychology is conducive to improve the management of AIBD and enhance quality of life among these patients.

## Data Availability

The datasets used and analysed during the current study are available from the corresponding author on reasonable request and can be deposited publically. Link of raw data: https://data.mendeley.com/datasets/73r8vrxksx

## Abbreviations

AIBD: Autoimmune blistering skin diseases
OSDI: Ocular surface disease index
SF-36: Medical Outcomes Study 36-Item Short Form Questionnaire
HADS: Hospital Anxiety and Depression Scale
PSQI: Pittsburgh Sleep Quality Index
HAQ-DI: Health Assessment Questionnaire-Disability Index
PV: Pemphigus vulgaris
PF: Pemphigus foliaceus
PNP: Paraneoplastic pemphigus
BP: Bullous pemphigoid
MMP: Mucous membrane pemphigoid
EBA: Epidermolysis bullosa acquisita
OCP: Ocular cicatricial pemphigoid
MGD: Meibomian gland dysfunction
TO: Tear osmolality
TBUT: Tear break-up time
LIPCOF: Lid-parallel conjunctival folds
PCS: Physical component summary
MCS: Mental component summary
